# Disproportionate Impact of COVID-19 Pandemic on Racial and Ethnic Minorities

**DOI:** 10.1177/0003134820973356

**Published:** 2020-12

**Authors:** Brad Boserup, Mark McKenney, Adel Elkbuli

**Affiliations:** 1 Department of Surgery, Division of Trauma and Surgical Critical Care, 14506 Kendall Regional Medical Center, Miami, FL, USA; 2 Department of Surgery, University of South Florida, Tampa, FL, USA

**Keywords:** COVID-19, racial disparities, health disparities, social determinants of health

## Abstract

**Background:**

Health disparities are prevalent in many areas of medicine. We aimed to investigate the impact of the COVID-19 pandemic on racial/ethnic groups in the United States (US) and to assess the effects of social distancing, social vulnerability metrics, and medical disparities.

**Methods:**

A cross-sectional study was conducted utilizing data from the COVID-19 Tracking Project and the Centers for Disease Control and Prevention (CDC). Demographic data were obtained from the US Census Bureau, social vulnerability data were obtained from the CDC, social distancing data were obtained from Unacast, and medical disparities data from the Center for Medicare and Medicaid Services. A comparison of proportions by Fisher’s exact test was used to evaluate differences between death rates stratified by age. Negative binomial regression analysis was used to predict COVID-19 deaths based on social distancing scores, social vulnerability metrics, and medical disparities.

**Results:**

COVID-19 cumulative infection and death rates were higher among minority racial/ethnic groups than whites across many states. Older age was also associated with increased cumulative death rates across all racial/ethnic groups on a national level, and many minority racial/ethnic groups experienced significantly greater cumulative death rates than whites within age groups ≥ 35 years. All studied racial/ethnic groups experienced higher hospitalization rates than whites. Older persons (≥ 65 years) also experienced more COVID-19 deaths associated with comorbidities than younger individuals. Social distancing factors, several measures of social vulnerability, and select medical disparities were identified as being predictive of county-level COVID-19 deaths.

**Conclusion:**

COVID-19 has disproportionately impacted many racial/ethnic minority communities across the country, warranting further research and intervention.

## Introduction

The initial outbreak of COVID-19 caused by severe acute respiratory syndrome coronavirus 2 (SARS-CoV-2) occurred during December 2019 in Wuhan, China.^1^ Since then, SARS-CoV-2 has spread rapidly, and on March 11, 2020, COVID-19 was characterized as a pandemic by the World Health Organization.^2^ By July 24, 2020, there were 4 024 492 confirmed cases and 143 868 deaths in the United States alone.^3^ SARS-CoV-2 is primarily transmitted person-to-person via respiratory droplets and is highly contagious with an average basic reproduction number (R0) of 3.28.^4^ The incubation period lasts for approximately 5.2 days, after which patients typically experience a fever, cough, and shortness of breath.^5^ While many patients with COVID-19 develop only mild disease, approximately 14% progress to a severe state (hypoxia, tachypnea, or >50% lung infiltrates), and approximately 5% progress to a critical state (shock, respiratory failure, or multiorgan failure).^6^

Severe disease can occur in healthy individuals of all ages; however, older persons with preexisting comorbidities and racial/ethnic minority groups appear to be most at risk.^7,8^ For instance, a report published by the Centers for Disease Control and Prevention (CDC) that examined laboratory-confirmed COVID-19 hospitalizations in 14 states found that blacks were 1.8 times more likely to be affected by COVID-19 than would be expected based on population data alone.^9^ These findings are further substantiated by state health department data that suggest blacks have been disproportionally affected by COVID-19 in a number of states across the United States ^10,11^ However, the impact of COVID-19 on other racial/ethnic minorities in the United States at the state and county levels remains largely uninvestigated since disaggregated data according to race and ethnicity are just recently becoming available. Therefore, further research is required to elucidate the impact COVID-19 has had on various racial/ethnic groups. The primary objective of this study is to identify how COVID-19 has affected all major racial/ethnic groups in the United States on a county, state, and national level. The modifying effects of social distancing, social vulnerability metrics, and medical disparities will also be examined.

## Methods

### Data Source and Population

#### COVID-19 data

This cross-sectional study was conducted utilizing publicly available cumulative case and death data from the COVID-19 Tracking Project^12^ (data updated as of July 15, 2020), which aggregates data from state departments of health. The COVID-19 data obtained from the COVID-19 Tracking Project included state-level data from the 48 states/regions that reported COVID-19 case and death data according to race and ethnicity at the time of writing (Supplementary Table 1 and Supplementary Table 2). Nationally aggregated cumulative data for COVID-19 deaths according to age, race, and ethnicity were obtained from the CDC (data included information from all states/regions in the United States and was recorded from the week of February 1, 2020-the week of July 11, 2020).^13^ Cumulative county-level COVID-19 death data according to race and ethnicity were obtained from the CDC for counties with more than 100 COVID-19 deaths. This data set included 173 counties spanning 37 states at the time of writing (data updated as of July 15, 2020) (Supplementary Table 3).^14^

Cumulative laboratory-confirmed COVID-19 associated hospitalization data was obtained from the CDC's COVID-19-Associated Hospitalization Surveillance Network (COVID-NET), which recorded the data from March 1, 2020, to July 11, 2020 (This data from the COVID-NET includes information from approximately 100 counties across 14 states: California, Colorado, Connecticut, Georgia, Iowa, Maryland, Michigan, Minnesota, New Mexico, New York, Ohio, Oregon, Tennessee, Utah).^15^ Cumulative nationally aggregated COVID-19 death data according to comorbidity status and age were also collected from the CDC (data included information from all states/regions in the United States and was recorded from the week of February 1, 2020-July 11, 2020).^13^

### Social Distancing, Social Vulnerability, and Medical Disparities Data

Social distancing scores at the county-level were obtained from Unacast. Unacast generates social distancing scores by tracking mobile phone interactions and were updated as of July 9, 2020.^16^ County-level social vulnerability data for unemployment rates, percentage of persons aged ≥65 years, percentage of persons ≥ age of 5 years who speak English “less than well,” percentage of occupied housing units with more people than rooms, percentage of households with no vehicle available, and percentage of persons in poverty in the 90th percentile were obtained from the CDC’s 2018 social vulnerability index data set.^17^ The percentage of white, black, Asian, and Hispanic Medicare beneficiaries with select conditions [chronic obstructive pulmonary disease (COPD), congestive heart failure, diabetes, or hypertension] at the county-level was obtained from the Centers for Medicare and Medicaid Services Mapping Medical Disparities Tool (data were updated as of June 29, 2020).^18^

### Census Data

Data were obtained from the US Census Bureau American Community Survey 5-Year Estimate.^19^ Census data were acquired at the country-level and state-level for the racial and ethnic groups studied [white, black, Hispanic or Latino, Asian, Native Hawaiian and Pacific Islanders (NHPIs), and American Indian and Alaska Native (AIAN)].

### Data Analysis and Statistical Methods

COVID-19 cases and deaths according to race and ethnicity were converted to rates/100 000 population using census data. A simple comparison of proportions by Fisher’s exact test was used to evaluate if each minority group (black, Hispanic or Latino, Asian, NHPI, and AIAN) experienced significantly different death rates on a national level (stratified by age) compared to whites. Negative binomial regression was used to evaluate the explanatory effect of social distancing, social vulnerabilities, and medical disparities on county-level ethnic/minority COVID-19 deaths/100 000 population. IBM SPSS statistical software version 27.0 (Armonk, New York) was used for data analysis. Statistical significance was defined as *P* < .05.

## Results

### State Reported COVID-19 Cases and Deaths According to Race and Ethnicity

Regarding nationwide infection rates, blacks were most affected compared to whites in Maine (4619/100 000; rate 27.0 times higher than whites), and Hispanics were most affected compared to whites in New Mexico (267 cases/100 000; rate 2.1 times higher than whites). Asians were most affected compared to whites in South Dakota (5656 cases/100 000; rate 14.7 times higher than whites), while AIAN individuals were most affected compared to whites in New Mexico (3396 cases/100 000; rate 26.1 times higher than whites). NHPI individuals were most affected compared to whites in Arkansas (22 989 cases/100 000; rate 36.1 times higher than whites) (Supplementary Table 1). Regarding nationwide death rates, blacks were most affected compared to whites in the District of Columbia (132 deaths/100 000; rate 6.3 times higher than whites). Asians were most affected compared to whites in Utah (16 deaths/100 000; rate 4.0 times higher than whites). AIANs were most affected compared to whites in Wyoming (64 deaths/100 000; rate 32.0 times higher than whites), and NHPIs were most affected compared to whites in Arkansas (364 deaths/100 000; rate 45.5 times higher than whites), while Hispanics were not disproportionately affected (Supplementary Table 2).

### National Reported COVID-19 Deaths According to Age, Race, and Ethnicity

**Figure 1.**
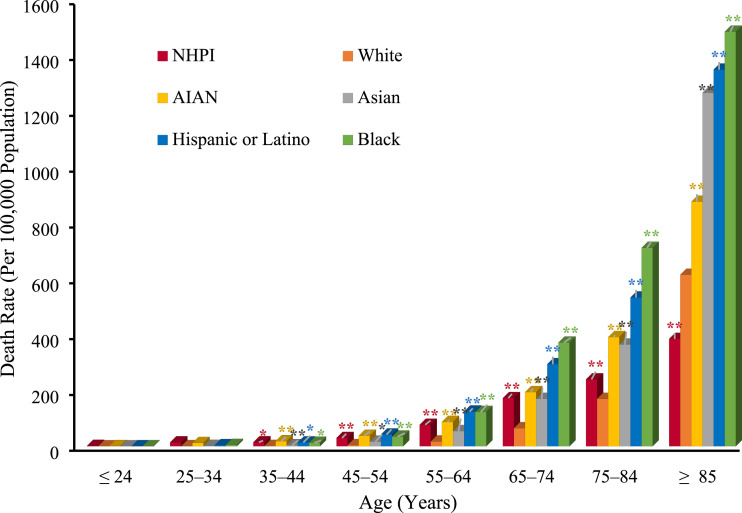
Cumulative crude COVID-19 death rates (per 100 000 population) according to age, race, and ethnicity (data recorded by the Centers for Disease Control from the week of February 1, 2020-the week of July 11, 2020). Asterisks indicate a significant difference in comparison to whites within each age group studied. Significance was determined via a simple comparison of proportions by Fisher’s exact test. *P < .01; **P < .001.

Older age was associated with increased cumulative death rates across all racial/ethnic groups on a national level. When stratified by age, blacks, Hispanic/Latinos, Asians, and AIANs consistently experienced significantly (*P* < .05) greater cumulative death rates (from February 1, 2020-the week of July 11, 2020) than whites within age groups ≥ 35 years (35-44, 45-54, 55-64, 65-74, 75-84, and ≥ 85 years) (Figure 1). Conversely, NHPI individuals in the ≥85 years group experienced significantly (*P* < .05) less deaths/100 000 compared to whites.

### Hospitalization Rates and Comorbidities

**Figure 2.**
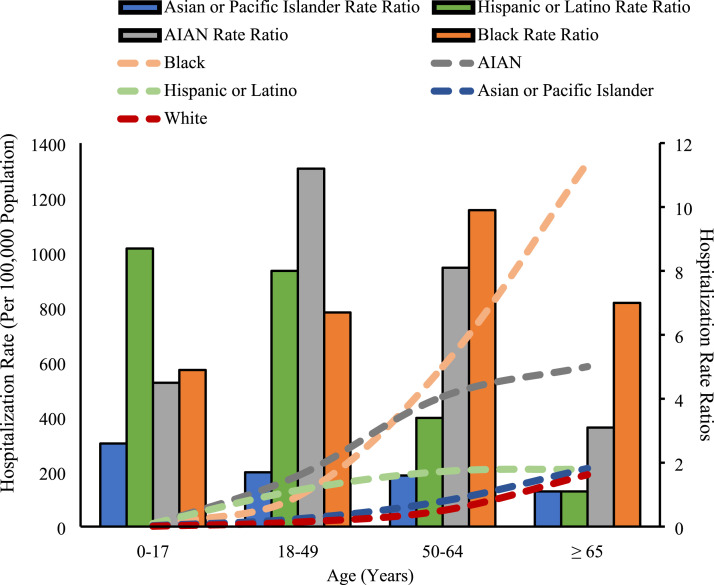
Cumulative crude laboratory-confirmed COVID-19-associated hospitalizations rates (data represent the 14 states in the COVID-NET network and were recorded from March 1, 2020-July 11, 2020). The dashed lines represent hospitalization rates (per 100 000 population) according to age, race, and ethnicity while the bar graph represents hospitalization rate ratios according to age, race, and ethnicity. Rate ratios are the ratio of racial/ethnic cumulative crude COVID-19-associated hospitalizations rates to age-matched white cumulative crude COVID-19-associated hospitalizations rates.

**Figure 3.**
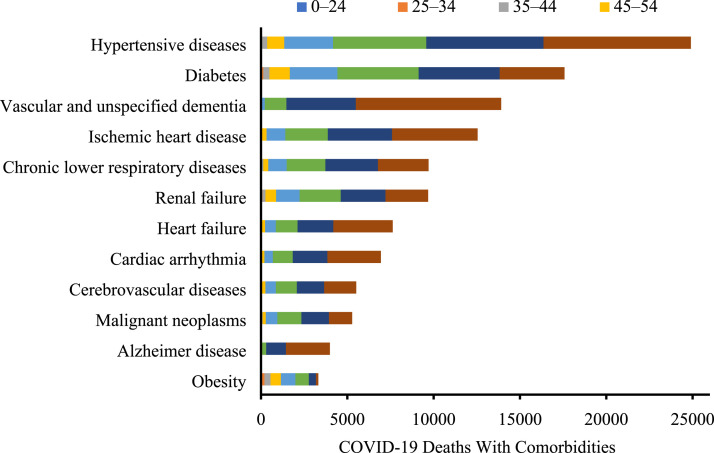
Cumulative COVID-19 deaths according to comorbidity and age (data were collected by the Centers for Disease Control and Prevention from February 1, 2020-July 11, 2020).

Blacks, Hispanic/Latinos, Asians or Pacific Islanders, and AIANs all experienced higher hospitalization rates than whites within age-stratified groups. Hispanic/Latinos had the highest hospitalization rate ratio (ratio of racial/ethnic cumulative crude COVID-19-associated hospitalizations rates to white cumulative crude COVID-19-associated hospitalizations rates) in the 0-17 years age group (8.7 times higher than whites). AIAN individuals had the highest hospitalization rate ratio in the 18-49 years age group (11.2 times higher than whites), and black individuals had the highest hospitalization rate ratio in both the 50-64 years (9.9 times higher than whites) and the ≥65 years age groups (7.0 times higher than whites) (Figure 2). Older persons (≥65 years) also experienced substantially more COVID-19 deaths associated with comorbidities compared to younger individuals (Figure 3).

### The Impact of Social Distancing, Social Vulnerability, and Medical Disparities

Results of the negative binomial regression models predicting county-level COVID-19 associated deaths/100 000 population based on social distancing scores, measures of social vulnerability, and race/ethnicity-specific comorbidity prevalence can be found in Table 1. The predicted number of white COVID-19 deaths/100 000 population was significantly higher in counties with an increased percentage of households without a vehicle (Incidence Rate Ratio (IRR) = 1.04, 95% CI: 1.02-1.05, *P* < .001) and significantly higher in counties with an increased percentage of white Medicare beneficiaries with diabetes (IRR = 1.07, 95% CI: 1.02-1.12, *P* = .004).

**Table 1.** Negative Binomial Regression Analysis Models Predicting County-Level White, Black, Asian, and Hispanic COVID-19-Associated Deaths/100 000 Population Based on Social Distancing Scores, Measures of Social Vulnerability, and Race/Ethnicity Specific Comorbidity Prevalence.

Variable	White			Black			Asian			Hispanic		
	IRR	*P*	95% CI	IRR	*P*	95% CI	IRR	*P*	95% CI	IRR	*P*	95% CI
Unacast social distancing score	.99	.951	[.67, 1.45]	.48	.033	[.24, .94]	.11	.044	[.01, .94]	1.46	.222	[.80, 2.70]
Unemployment rate estimate	.96	.374	[.88, 1.05]	.84	.020	[.73, .97]	.64	.012	[.46, .91]	.86	.022	[.76, .98]
Percentage of persons aged 65 years and older	1.04	.074	[1.00, 1.09]	.94	.134	[.87, 1.02]	.92	.395	[.77, 1.11]	1.06	.119	[.99, 1.14]
Percentage of persons (age 5 years+) who speak English “less than well”	1.01	.825	[.95, 1.07]	.98	.676	[.89, 1.08]	1.13	.297	[.90, 1.42]	1.22	<.001	[1.10, 1.34]
Percentage of occupied housing units with more people than rooms	.85	.053	[.73, 1.00]	.89	.121	[.77, 1.03]	1.07	.760	[.70, 1.62]	.93	.381	[.80, 1.09]
Percentage of households with no vehicle available	1.04	<.001	[1.02, 1.05]	1.05	<.001	[1.03, 1.08]	1.12	.002	[1.04, 1.20]	1.03	.022	[1.004, 1.052]
Percentage of persons in poverty is in the 90th percentile	1.31	.439	[.67, 2.56]	2.79	.126	[.75, 10.34]	.20	.250	[.01, 3.07]	.78	.669	[.25, 2.47]
Percentage of race/ethnicity specific Medicare beneficiaries with COPD	.96	.213	[.89, 1.03]	1.02	.661	[.92, 1.14]	.68	.032	[.48, .97]	.89	.023	[.81, .98]
Percentage of race/ethnicity specific Medicare beneficiaries with CHF	.99	.766	[.92, 1.06]	.97	.537	[.87, 1.08]	1.12	.512	[.80, 1.57]	1.01	.799	[.92, 1.12]
Percentage of race/ethnicity specific Medicare beneficiaries with diabetes	1.07	.004	[1.02, 1.12]	1.12	.002	[1.04, 1.20]	1.09	.197	[.96, 1.25]	1.18	<.001	[1.11, 1.25]
Percentage of race/ethnicity specific Medicare beneficiaries with hypertension	1.02	.223	[1.00, 1.05]	1.06	.069	[1.00, 1.13]	1.10	.220	[.94, 1.28]	.97	.196	[.93, 1.02]

Abbrevations: IRR, incidence rate ratio; CI, confidence Interval; COPD, chronic obstructive pulmonary disease; CHF, congestive heart failure.

The predicted number of black COVID-19 deaths/100 000 population was significantly higher in counties with an increased percentage of households without a vehicle (IRR = 1.05, 95% CI: 1.03-1.08, *P* < .001) and significantly higher in counties with an increased percentage of black Medicare beneficiaries with diabetes (IRR = 1.12, 95% CI: 1.04-1.20, *P* = .002). Conversely, the predicted number of black COVID-19 deaths/100 000 population was significantly lower in counties that had better social distancing scores (IRR = .48, 95% CI: .24-.94, *P* = .033) and significantly lower in counties with higher unemployment rates (IRR = .84, 95% CI: .73-.97, *P* = .020).

The predicted number of Asian COVID-19 deaths/100 000 population was significantly higher in counties with an increased percentage of households without a vehicle (IRR = 1.12, 95% CI: 1.04-1.20, *P* = .002). Conversely, the predicted number of Asian COVID-19 deaths/100 000 population was significantly lower in counties that had better social distancing scores (IRR = .11, 95% CI: .01-.94, *P* = .044), was significantly lower in counties with higher unemployment rates (IRR = .64, 95% CI: .46-.91, *P* = .012), and was significantly lower in counties with an increased percentage of Asian Medicare beneficiaries with COPD (IRR = .68, 95% CI: .48-.97, *P* = .032).

The predicted number of Hispanic COVID-19 deaths/100 000 population was significantly higher in counties with an increased percentage of persons (age ≥5 years) who speak English “less than well” (IRR = 1.22, 95% CI: 1.10-1.34, *P* < .001), was significantly higher in counties with an increased percentage of households with no vehicle available (IRR = 1.03, 95% CI: 1.004-1.052, *P* = .022), and was significantly higher in counties with an increased percentage of Hispanic Medicare beneficiaries with diabetes (IRR = 1.18, 95% CI: 1.11-1.25, *P* < .001). Additionally, the predicted number of Hispanic COVID-19 deaths/100 000 population was significantly lower in counties with higher unemployment rates (IRR = .86, 95% CI: .76-.98, *P* < .022) and was significantly lower in counties with an increased percentage of Hispanic Medicare beneficiaries with COPD (IRR = .89, 95% CI: .81-.98, *P* = .023).

## Discussion

All studied minority racial/ethnic groups (blacks, Hispanics, Asians, AIANs, NHPIs) experienced disproportionate infection rates compared to whites in many different states. However, with regard to death rates, blacks, Asians, AIANs, and NHPIs were disproportionally affected compared to whites, while Hispanics were not disproportionately affected. This unexpected finding may be due to inconsistent reporting of Hispanic/Latino status across state health departments, possibly leading to inaccurate comparisons since this anomaly was not observed in aggregated national data from the CDC.

Overall, older age was associated with increased death rates across all studied racial/ethnic groups. This observed disparity may be due to increased rates of comorbid conditions among minority groups.^20^ In particular, several chronic conditions prevalent among minority groups, such as hypertensive disease, diabetes, and ischemic heart disease, were frequently observed in COVID-19 deaths with associated comorbidities. Nonetheless, these findings demonstrate that older age only exacerbates underlying racial/ethnic disparities. Blacks, Hispanic/Latinos, Asians, and AIANs within the study age groups ≥35 years (35-44, 45-54, 55-64, 65-74, 75-84, and ≥85 years) were disproportionately affected compared to whites. In contrast, NHPIs in the ≥85 years group were less affected compared to whites. In the absence of disaggregated COVID-19 infection rates according to age and race, it is difficult to assess why NHPIs experienced lower death rates than whites since, as a whole, the population has been disproportionately impacted. However, this interesting finding may be partially due to the relatively young age of NHPI individuals residing in the United States (31% of NHPI individuals are <18 years old compared to 19% of the white population).^21^

Surprisingly, despite experiencing infection and death rates many times higher than whites experience, Hispanic/Latinos and Asians or Pacific Islanders were hospitalized at rates only slightly higher than whites were, with the smallest difference seen among older persons (≥65 years). These data indicate that disparities in access to quality care may be more prevalent among Hispanic/Latinos and Asians or Pacific Islanders compared to other populations. In addition, in addition to disparities in access to care, variation in the time taken to seek medical attention may be responsible for poorer outcomes among racial/ethnic minority groups. While not examined in this study, due to limited data availability, this potentially confounding factor should be investigated in future research.

A number of factors were also identified as being predictive of county-level COVID-19 deaths. Interestingly, an increased percentage of households with no vehicle available was predictive of all deaths regardless of race/ethnicity. This finding indicates that the use of public transportation is a potentially leading risk factor, and efforts should be taken to reduce COVID-19 transmission among people who rely on these services. An increased percentage of white, black, and Hispanic Medicare beneficiaries with diabetes was also associated with an increased number of county-level COVID-19 deaths among these respective racial/ethnic groups. Thus, these findings further corroborate existing evidence that suggests diabetes is a leading risk factor for worse COVID-19 outcomes.^22^ Paradoxically, an increased percentage of Asian and Hispanic Medicare beneficiaries with COPD was predictive of fewer county-level COVID-19 deaths. Since COPD is known to be associated with worse COVID-19 outcomes, it is possible that individuals with this condition are being extra cautious and are taking measures to avoid infection.^23^ Further research in this area would be helpful to understand this finding.

Additionally, better social distancing scores were only predictive of fewer black and Asian county-level COVID-19 deaths and were not predictive of white or Hispanic deaths. These observed differences in the effect of social distancing may be due to the fluctuating nature of this metric based on current events. For instance, individuals in some counties may take a more proactive approach to social distancing, while individuals in other counties may only start to social distance after death counts rise due to fear. Similar to social distancing, increased unemployment rates were also predictive of fewer black, Asian, and Hispanic county-level COVID-19 deaths. Thus, unemployment rates could potentially serve as a pseudo social distancing metric since being unemployed likely reduces exposure risk. Finally, an increased percentage of persons (age ≥5 years) who speak English “less than well” was predictive of an increased number of Hispanic county-level COVID-19 deaths. This finding is particularly impactful since it highlights that precautionary measures in the United States are perhaps not adequately disseminated in other languages.

This study has several limitations. First, inconsistencies and incomplete reporting of COVID-19 race/ethnicity data by state health departments limited our analysis. Also, the county-level COVID-19 death data from the CDC used in this study and the COVID-19-associated hospitalization data from the CDC’s COVID-NET used in this study did not include data from all 50 states, and therefore, may not be representative of the entire United States However, at the time of writing, more complete data sources have not been made publicly available.

## Conclusions

Overall, this study indicates that all major racial and ethnic minority groups have been disproportionally impacted by the COVID-19 pandemic. While older individuals are known to be at increased risk, this study demonstrates that older minority groups are also disproportionally affected. In addition, relatively low hospitalization rates among some racial and ethnic minority groups, combined with higher death rates, may indicate substantial disparities in access to care. Finally, a number of variables, such as social distancing factors, measures of social vulnerability, and medical disparities, were found to be predictive of county-level COVID-19 deaths. In light of these findings, it will be paramount that all racial/ethnic minorities are adequately represented in COVID-19 vaccine trials since response rates may vary among minority groups, limiting our ability to “flatten the curve.” Therefore, we recommend developing diversity programs to bolster the enrollment of diverse trial participants to more adequately represent the entire country. Finally, steps also need to be taken to increase minority access to care and access to COVID-19 related information.

## Supplemental Material

sj-pdf-1-asu-10.1177_0003134820973356 – Supplemental Material for Disproportionate Impact of COVID-19 Pandemic on Racial and Ethnic Minorities Click here for additional data file. Supplemental Material, sj-pdf-1-asu-10.1177_0003134820973356 for Disproportionate Impact of COVID-19 Pandemic on Racial and Ethnic Minorities by Brad Boserup, Mark McKenney and Adel Elkbuli in The American Surgeon

sj-pdf-2-asu-10.1177_0003134820973356 – Supplemental Material for Disproportionate Impact of COVID-19 Pandemic on Racial and Ethnic Minorities Click here for additional data file. Supplemental Material, sj-pdf-2-asu-10.1177_0003134820973356 for Disproportionate Impact of COVID-19 Pandemic on Racial and Ethnic Minorities by Brad Boserup, Mark McKenney and Adel Elkbuli in The American Surgeon

sj-pdf-3-asu-10.1177_0003134820973356 – Supplemental Material for Disproportionate Impact of COVID-19 Pandemic on Racial and Ethnic Minorities Click here for additional data file. Supplemental Material, sj-pdf-3-asu-10.1177_0003134820973356 for Disproportionate Impact of COVID-19 Pandemic on Racial and Ethnic Minorities by Brad Boserup, Mark McKenney and Adel Elkbuli in The American Surgeon
