# Supplementary material for: Disproportionate Impact of COVID-19 Pandemic on Racial and Ethnic Minorities
Source: Am Surg. 2020 Dec;86(12):1615–22. doi: 10.1177/0003134820973356 (PMC7691116; doi:10.1177/0003134820973356)
Supplement: sj-pdf-1-asu-10.1177_0003134820973356 – Supplemental Material for Disproportionate Impact of COVID-19 Pandemic on Racial and Ethnic Minorities [file sj-pdf-1-asu-10.1177_0003134820973356.pdf]

**eTable 1.** Represents cumulative crude COVID-19 infection rates by state per 100,000 population. The number in parentheses represents how many times as high the minority infection rate is compared to Whites. Asterisks represent the highest infection rate disparity compared to Whites for each racial/ethnic group in the US (data updated by the COVID-19 Tracking Project as of July 15, 2020).

| State/Region | Cases Per 100,000 Population | | | | | |
|---|---|---|---|---|---|---|
| | White | Black | Hispanic | Asian | AIAN | NHPI |
| Alabama | 580 | 1418 (2.4) | 102 (0.2) | 359 (0.6) | | |
| Alaska | 140 | 162 (1.2) | 13 (0.1) | 140 (1.0) | 191 (1.4) | 599 (4.3) |
| Arizona | 396 | 944 (2.4) | 437 (1.1) | 423 (1.1) | 2688 (6.8) | |
| Arkansas | 637 | 1398 (2.2) | 245 (0.4) | 966 (1.5) | 389 (0.6) | 22989 (36.1)* |
| California | 168 | 428 (2.5) | 312 (1.9) | 235 (1.4) | 163 (1.0) | 879 (5.2) |
| Colorado | 255 | 783 (3.1) | 234 (0.9) | 467 (1.8) | 347 (1.4) | 1434 (5.6) |
| Connecticut | 623 | 1658 (2.7) | 253 (0.4) | 434 (0.7) | 454 (0.7) | |
| Delaware | 648 | 1588 (2.5) | 368 (0.6) | 509 (0.8) | | |
| District of Columbia | 803 | 1690 (2.1) | 448 (0.6) | 636 (0.8) | 1355 (1.7) | 8529 (10.6) |
| Florida | 406 | 1289 (3.2) | 370 (0.9) | | | |
| Georgia | 493 | 1034 (2.1) | 177 (0.4) | 409 (0.8) | 197 (0.4) | 2018 (4.1) |
| Hawaii | 58 | 38 (0.7) | | 63 (1.1) | | 241 (4.2) |
| Idaho | 397 | 1430 (3.6) | 138 (0.3) | 467 (1.2) | 443 (1.1) | 1557 (3.9) |
| Illinois | 375 | 1443 (3.8) | 386 (1.0) | 633 (1.7) | 716 (1.9) | 8512 (22.7) |
| Indiana | 446 | 1019 (2.3) | 90 (0.2) | 516 (1.2) | | |
| Iowa | 848 | 2974 (3.5) | 255 (0.3) | 2902 (3.4) | 3143 (3.7) | 11601 (13.7) |
| Kansas | 548 | 933 (1.7) | 253 (0.5) | 1002 (1.8) | 595 (1.1) | |
| Kentucky | 284 | 523 (1.8) | 50 (0.2) | 740 (2.6) | 718 (2.5) | 1350 (4.8) |
| Maine | 171 | 4619 (27.0)* | 11 (0.1) | 583 (3.4) | 72 (0.4) | 629 (3.7) |
| Maryland | 456 | 1218 (2.7) | 330 (0.7) | 390 (0.9) | | |
| Massachusetts | 642 | 2066 (3.2) | 321 (0.5) | 531 (0.8) | | |
| Michigan | 408 | 1588 (3.9) | 63 (0.2) | 541 (1.3) | 579 (1.4) | |
| Minnesota | 410 | 2600 (6.3) | 165 (0.4) | 1136 (2.8) | 593 (1.4) | 4298 (10.5) |
| Mississippi | 617 | 1593 (2.6) | 57 (0.1) | 438 (0.7) | 8240 (13.4) | |
| Missouri | 268 | 1070 (4.0) | 73 (0.3) | | | |
| Montana | 130 | 259 (2.0) | 10 (0.1) | 63 (0.5) | 303 (2.3) | |
| Nebraska | 750 | 1431 (1.9) | 478 (0.6) | 2721 (3.6) | 1418 (1.9) | 2371 (3.2) |
| Nevada | 242 | 497 (2.1) | 228 (0.9) | 506 (2.1) | 271 (1.1) | |
| New Hampshire | 306 | 1531 (5.0) | 45 (0.1) | 448 (1.5) | | |
| New Jersey | 629 | 1506 (2.4) | 362 (0.6) | 643 (1.0) | | |
| New Mexico | 130 | 677 (5.2) | 267 (2.1)* | 346 (2.7) | 3396 (26.1)* | |
| North Carolina | 493 | 677 (1.4) | 258 (0.5) | 414 (0.8) | 575 (1.2) | 2781 (5.6) |
| Ohio | 347 | 1280 (3.7) | 42 (0.1) | 795 (2.3) | 352 (1.0) | 3704 (10.7) |
| Oklahoma | 448 | 590 (1.3) | 122 (0.3) | 1766 (3.9) | 541 (1.2) | |
| Oregon | 158 | 698 (4.4) | 121 (0.8) | 251 (1.6) | 603 (3.8) | 2187 (13.8) |
| Pennsylvania | 298 | 927 (3.1) | 61 (0.2) | 327 (1.1) | | |
| Rhode Island | 571 | 2352 (4.1) | 575 (1.0) | | | |
| South Carolina | 613 | 1119 (1.8) | 111 (0.2) | 605 (1.0) | | |
| South Dakota | 386 | 6077 (15.7) | 131 (0.3) | 5656 (14.7)* | 1641 (4.3) | |
| Tennessee | 563 | 1266 (2.2) | 220 (0.4) | 656 (1.2) | 510 (0.9) | 1498 (2.7) |
| Texas | 31 | 89 (2.9) | 38 (1.2) | 50 (1.6) | | |
| Utah | 443 | 2161 (4.9) | 411 (0.9) | 980 (2.2) | 2147 (4.8) | 4451 (10.0) |
| Vermont | 184 | 1812 (9.8) | 8 (0.0) | 501 (2.7) | 95 (0.5) | |
| Virginia | 281 | 707 (2.5) | 283 (1.0) | 435 (1.5) | 379 (1.3) | |
| Washington | 198 | 611 (3.1) | 182 (0.9) | 270 (1.4) | 508 (2.6) | 1844 (9.3) |
| West Virginia | 212 | 486 (2.3) | | | | |
| Wisconsin | 505 | 1831 (3.6) | 193 (0.4) | 908 (1.8) | 762 (1.5) | |
| Wyoming | 175 | 361 (2.1) | 44 (0.3) | 189 (1.1) | 2462 (14.1) | 1113 (6.4) |
