# Supplementary material for: Disproportionate Impact of COVID-19 Pandemic on Racial and Ethnic Minorities
Source: Am Surg. 2020 Dec;86(12):1615–22. doi: 10.1177/0003134820973356 (PMC7691116; doi:10.1177/0003134820973356)
Supplement: sj-pdf-2-asu-10.1177_0003134820973356 – Supplemental Material for Disproportionate Impact of COVID-19 Pandemic on Racial and Ethnic Minorities [file sj-pdf-2-asu-10.1177_0003134820973356.pdf]

eTable 2. Represents crude COVID-19 death rates by state per 100,000 population. The number in parentheses represents how many times as high the minority death rate is compared to Whites. Asterisks represent the highest death rate disparity compared to Whites for each racial/ethnic group in the US (data updated by the COVID-19 Tracking Project as of July 15, 2020).

State	Deaths Per 100,000 Population					
	White	Black	Hispanic	Asian	AIAN	NHPI
Alabama	18	40 (2.2)	1 (0.1)	6 (0.3)		
Alaska	2	0 (0.0)	0 (0.0)	4 (2.0)	3 (1.5)	11 (5.5)
Arizona	18	23 (1.3)	9 (0.5)	12 (0.7)	114 (6.3)	
Arkansas	8	19 (2.4)	1 (0.1)	14 (1.8)	0 (0.0)	364 (45.5)*
California	9	27 (3.0)	8 (0.9)*	17 (1.9)	8 (0.9)	22 (2.4)
Colorado	21	47 (2.2)	6 (0.3)	31 (1.5)	15 (0.7)	60 (2.9)
Connecticut	117	171 (1.5)	10 (0.1)	30 (0.3)	21 (0.2)	
Delaware	50	64 (1.3)	3 (0.1)	3 (0.1)		
District of Columbia	21	132 (6.3)*	11 (0.5)	30 (1.4)		
Florida	14	27 (1.9)	6 (0.4)			
Georgia	23	44 (1.9)	2 (0.1)	12 (0.5)	12 (0.5)	98 (4.3)
Idaho	7	9 (1.3)	0 (0.0)	8 (1.1)	4 (0.6)	
Illinois	35	110 (3.1)	12 (0.3)	50 (1.4)	31 (0.9)	175 (5.0)
Indiana	30	60 (2.0)	1 (0.0)	9 (0.3)		
Iowa	22	35 (1.6)	2 (0.1)	31 (1.4)	61 (2.8)	479 (21.8)
Kansas	8	38 (4.8)	1 (0.1)	11 (1.4)	8 (1.0)	
Kentucky	13	26 (2.0)	1 (0.1)	13 (1.0)	0 (0.0)	
Louisiana	51	111 (2.2)	2 (0.0)	29 (0.6)	15 (0.3)	341 (6.7)
Maine	8	11 (1.4)	0 (0.0)			
Maryland	42	76 (1.8)	6 (0.1)	35 (0.8)		
Massachusetts	117	134 (1.1)	8 (0.1)	49 (0.4)		
Michigan	42	182 (4.3)	1 (0.0)	26 (0.6)	36 (0.9)	
Minnesota	26	44 (1.7)	1 (0.0)	25 (1.0)	49 (1.9)	137 (5.3)
Mississippi	30	57 (1.9)	1 (0.0)	0 (0.0)	541 (18.0)	
Missouri	13	54 (4.2)	0 (0.0)			
Montana	2				15 (7.5)	
Nebraska	13	24 (1.8)	4 (0.3)	36 (2.8)	41 (3.2)	0 (0.0)
Nevada	14	26 (1.9)	3 (0.2)	35 (2.5)	11 (0.8)	
New Hampshire	26	39 (1.5)	1 (0.0)	8 (0.3)		
New Jersey	120	205 (1.7)	30 (0.3)	89 (0.7)		
New Mexico	6	9 (1.5)	3 (0.5)	3 (0.5)	118 (19.7)	
New York	68	206 (3.0)	34 (0.5)	104 (1.5)		
North Carolina	12	23 (1.9)	1 (0.1)	8 (0.7)	19 (1.6)	43 (3.6)
Ohio	25	41 (1.6)	1 (0.0)	13 (0.5)	4 (0.2)	28 (1.1)
Oklahoma	11	10 (0.9)	0 (0.0)	10 (0.9)	12 (1.1)	
Oregon	5	8 (1.6)	1 (0.2)	5 (1.0)	6 (1.2)	37 (7.4)
Pennsylvania	45	100 (2.2)	3 (0.1)	35 (0.8)		
Rhode Island	77	74 (1.0)	8 (0.1)			
South Carolina	14	31 (2.2)	1 (0.1)	12 (0.9)		
Tennessee	8	25 (3.1)	1 (0.1)	12 (1.5)	0 (0.0)	26 (3.3)
Texas	2	3 (1.5)	1 (0.5)	1 (0.5)		
Utah	4	14 (3.5)	2 (0.5)	16 (4.0)*	64 (16.0)	48 (12.0)
Vermont	9		0 (0.0)	19 (2.1)		
Virginia	19	29 (1.5)	3 (0.2)	22 (1.2)	9 (0.5)	
Washington	17	18 (1.1)	3 (0.2)	19 (1.1)	32 (1.9)	29 (1.7)
West Virginia	3	4 (1.3)				
Wisconsin	12	53 (4.4)	2 (0.2)	14 (1.2)	20 (1.7)	
Wyoming	2	0 (0.0)	0 (0.0)	0 (0.0)	64 (32.0)*	
