# Supplementary material for: Disproportionate Impact of COVID-19 Pandemic on Racial and Ethnic Minorities
Source: Am Surg. 2020 Dec;86(12):1615–22. doi: 10.1177/0003134820973356 (PMC7691116; doi:10.1177/0003134820973356)
Supplement: sj-pdf-3-asu-10.1177_0003134820973356 – Supplemental Material for Disproportionate Impact of COVID-19 Pandemic on Racial and Ethnic Minorities [file sj-pdf-3-asu-10.1177_0003134820973356.pdf]

eTable 3. Counties with more than 100 COVID-19 deaths in the US (data updated as of July 15, 2020).

State	Counties
Alabama	Jefferson, Mobile, Montgomery
Arizona	Coconino, Maricopa, Pima, Yuma
California	Alameda, Los Angeles, Orange, Riverside, San Bernardino, San Diego, San Mateo, Santa Clara, Tulare
Colorado	Adams, Arapahoe, Denver, El Paso, Jefferson, Weld
Connecticut	Fairfield, Hartford, Litchfield, Middlesex, New Haven
Delaware	New Castle, Sussex
District of Columbia	District of Columbia
Florida	Broward, Hillsborough, Lee, Manatee, Miami-Dade, Palm Beach, Pinellas, Polk
Georgia	Cobb, DeKalb, Dougherty, Fulton, Gwinnett
Illinois	Cook, DuPage, Kane, Lake, St. Clair, Will, Winnebago
Indiana	Allen, Hendricks, Lake, Marion
Iowa	Polk
Kentucky	Jefferson
Louisiana	Caddo Parish, East Baton Rouge Parish, Jefferson Parish, Orleans Parish, St. Tammany Parish
Maryland	Anne Arundel, Baltimore, Carroll, Frederick, Montgomery, Prince George's, Baltimore city
Massachusetts	Barnstable, Bristol, Essex, Hampden, Middlesex, Norfolk, Plymouth, Suffolk, Worcester
Michigan	Genesee, Kent, Macomb, Oakland, Saginaw, Washtenaw, Wayne
Minnesota	Anoka, Hennepin, Ramsey
Mississippi	Hinds
Missouri	St. Louis, St. Louis city
Nebraska	Douglas
Nevada	Clark
New Hampshire	Hillsborough
New Jersey	Atlantic, Bergen, Burlington, Camden, Cumberland, Essex, Gloucester, Hudson, Hunterdon, Mercer, Middlesex, Monmouth, Morris, Ocean, Passaic, Somerset, Sussex, Union, Warren
New Mexico	Bernalillo, San Juan
New York	Albany, Bronx, Dutchess, Erie, Kings, Monroe, Nassau, New York, Oneida, Onondaga, Orange, Queens, Richmond, Rockland, Suffolk, Westchester
Ohio	Cuyahoga, Franklin, Hamilton, Lucas, Mahoning, Stark, Summit
Oklahoma	Tulsa
Pennsylvania	Allegheny, Berks, Bucks, Chester, Dauphin, Delaware, Lackawanna, Lancaster, Lehigh, Luzerne, Montgomery, Northampton, Philadelphia
Rhode Island	Providence
South Carolina	Florence, Richland
Tennessee	Davidson, Shelby
Texas	Bexar, Dallas, El Paso, Galveston, Harris, Tarrant, Travis
Utah	Salt Lake
Virginia	Arlington, Fairfax, Henrico, Loudoun
Washington	King, Snohomish, Yakima
Wisconsin	Milwaukee
